# Ascending sensory motor polyradiculoneuropathy with cranial nerve involvement following administration of intrathecal methotrexate and intravenous cytarabine in a patient with acute myelogenous leukemia: a case report*

**DOI:** 10.1186/1757-1626-1-255

**Published:** 2008-10-21

**Authors:** Richard A Rison

**Affiliations:** 1 Whittier Presbyterian Intercommunity Hospital Stroke Center, University of Southern California, Keck School of Medicine, Los Angeles County Medical Center, Neurology Consultants Medical Group, Whittier, California, USA

## Abstract

**Background:**

Acute inflammatory polyradiculoneuropathy secondary to chemotherapy for leukemia has been described in the pediatric literature. However, the reports are rare and have been mainly from intrathecal methotrexate in pediatric acute lymphoblastic leukemia patients who developed demyelinating polyradiculoneuropathy.

**Case presentation:**

A case report is presented of an unfortunate 53 year old Hispanic woman with acute myelogenous leukemia who developed profound weakness with cranial nerve palsies following both intravenous and intrathecal chemotherapy.

**Conclusion:**

This is an interesting and unusual case of predominantly axonal ascending sensory motor polyradiculoneuropathy with cranial nerve involvement in an adult patient with acute myelogenous leukemia following intravenous Cytosine arabinoside and intrathecal methotrexate.

## Background

Polyradiculoneuropathies and ascending motor paraplegia have been described in pediatric leukemia patients undergoing chemotherapy. These rare reports have focused mainly on demyelinating polyradiculoneuropathies and/or myelopathies secondary to intrathecal methotrexate in children with acute lymphoblastic leukemia (ALL) [1]. A case report is presented of sensory motor axonal polyradiculoneuropathy with ascending paralysis and cranial nerve involvement in an adult patient with acute myelogenous leukemia (AML) following both intrathecal methotrexate and intravenous Cytosine arabinoside (ARA-C).

## Case history

**Figure 1:**
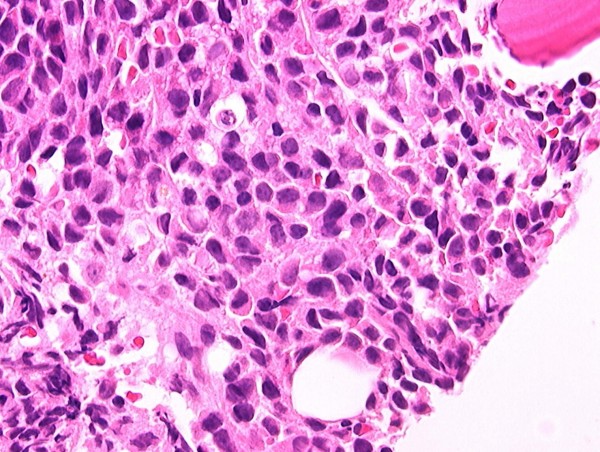
Bone marrow core biopsy demonstrating markedly increased cellularity with sheets of leukemic blasts morphologically consistent with myelomonocytic leukemic blasts.

The patient was a 53 year old Hispanic woman who originally presented at the end May in 2007 with complaints of shoulder tightness and weakness in the neck and shoulders for approximately 3 weeks duration along with shortness of breath. She presented to a local community hospital where examination revealed an ill-appearing pale woman with tachypnia. Subsequent investigations showed thrombocytopenia with prominent leukocytosis and numerous blasts on peripheral smear plus disseminated intravascular coagulation with subsequent diagnosis of acute myelogenous leukemia (please see Figure 1). She underwent one session of leukophoresis and was started on chemotherapy with Idarubicin (for the first 3 days) and ARA-C (for the first 7 days) along with allopurinol as tumor lysis prophylaxis and prophylactic intrathecal methotrexate. Shortly after her initial presentation and chemotherapy she suffered a left frontal lobe ischemic stroke with right hemiparesis and aphasia. After she responded to the leukophoresis and chemotherapy and made some neurologic improvement with physical therapy, she was transferred to the transitional care unit where she made dramatic improvements in her speech and balance and was able to walk up and down the hallway.

Approximately one month after her initial presentation she began losing strength in her right leg. This was followed by increased weakness in her right arm. A repeat MRI of the brain showed no evidence of a new stroke. Over the next three days she then went on to develop weakness in all four extremities plus dysphasia and respiratory difficulties, so that by the first weekend in July she was essentially quadriplegic. Magnetic resonance imaging studies of her cervical and lumbar spines showed no findings that could explain all of her symptoms. She then progressed to respiratory failure requiring intubation.

Neurologic evaluation revealed diffuse severe weakness of all extremities (1/4) with hypoactive reflexes (0/4). Extra ocular muscles were intact without dysconjugate gaze, and the pupils were briskly reactive and symmetric.

A lumbar puncture did not reveal any increased protein (33 mg/dL with normal range 20–40 mg/dL) or abnormal cells (red blood cells were 41 with normal < 5/cmm and white blood cells 3 with normal 0–5/cmm), however there were oligoclonal bands (36 with normal < 4) and an elevated myelin basic protein (4.3 with normal range 0.07–4.10 ng/mL). Cerebrospinal fluid cytology was negative for any malignant cells. An electromyogram and nerve conduction study showed absent sensory nerve action potentials and absent compound motor action potentials with diffuse denervation in all four extremities.

The presumptive diagnosis was Guillain-Barré syndrome (GBS), and intravenous immunoglobulin (IVIG) was considered but felt to be contraindicated secondary to her newly diagnosed AML and stroke. She was therefore given 4 sessions of plasmapharesis which was held on the fifth session because of blood pressure fluctuations.

Over the next ten days or so she did not improve neurologically, and in fact went on to develop right ptosis with a dysreactive pupil and outward eye deviation thought to be a third cranial nerve (CN 3) palsy, and a few days later bilateral facial weakness. A repeat lumbar puncture (about 2 weeks after the last one) again did not reveal any increased protein (27 mg/dL with normal range 20–40 mg/dL) or abnormal white blood cells (white blood cells were 0/cmm and red blood cells were 1/cmm) and the cerebrospinal fluid cytology was negative for any malignant cells.

A sensory-motor neuropathy panel, myasthenia gravis panel, and a Lambert-Eaton myasthenic panel both were negative for any abnormal antibodies (Quest Diagnostics).

**Figure 2:**
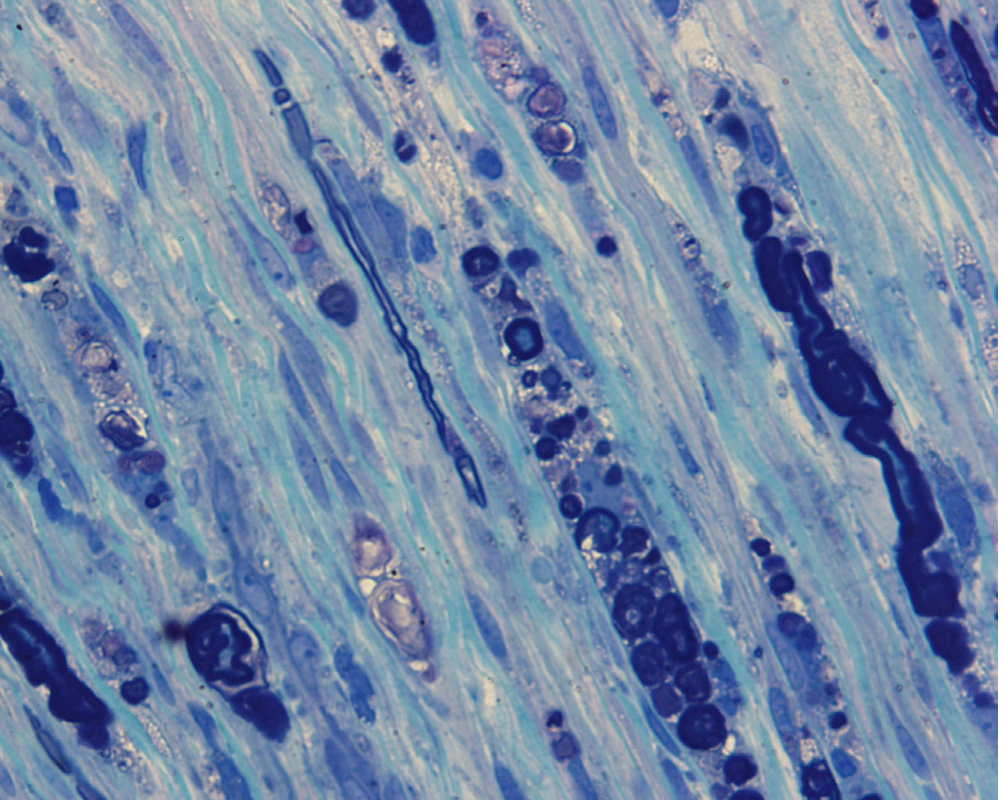
Sural nerve (Epon) section demonstrating evidence of axonal degeneration with digestion chambers and secondary dropout of myelinated fibers.

**Figure 3:**
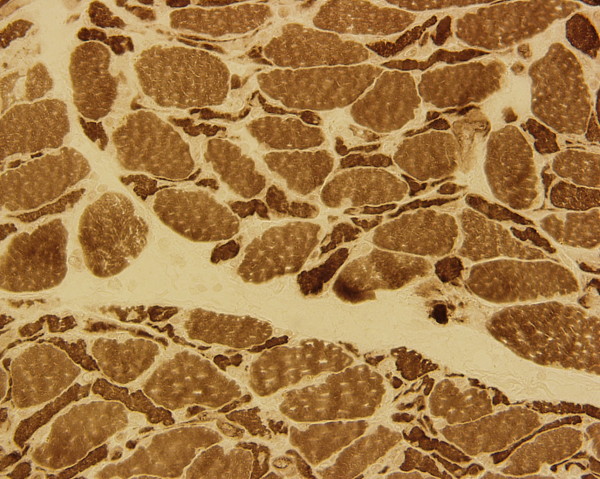
Muscle biopsy (ATPase stain) of the right thigh showing diffuse Type II fiber atrophy and focal myofiber degeneration.

A combined muscle and nerve biopsy was performed. The sural nerve showed peripheral neuropathy with prominent evidence of axonal degeneration with digestion chambers and secondary dropout of myelinated fibers which was moderately severe (please see Figure 2). There was no evidence of vasculitis nor of endoneurial inflammation. There was no evidence of amyloid deposition nor of hypertrophic neuropathy. The muscle biopsy (right thigh) showed diffuse Type II fiber atrophy and focal myofiber degeneration (please see Figure 3).

The patient remained on a ventilator and essentially quadriplegic with a CN 3 palsy and bilateral facial weakness. Approximately four weeks later she expired from pneumonia and secondary sepsis.

## Discussion

The incidence of acute inflammatory demyelinating polyradiculoneuropathy (AIDP) ranges from 0.6 to 2.4 cases per 100,000 population per year [2]. Cranial nerve involvement ranges from 45–75% in different series. Facial paresis, usually bilateral, is found in at least one half of patients. Involvement of extraocular muscles and lower cranial nerves is seen less often. The proportion of patients developing respiratory failure and requiring assisted ventilation seems to increase with age and ranges from 12% in epidemiological series to 30% in hospital-based series [3].

Although the most common neurotoxic effects of intravenous ARA-C involve the central nervous system, the association of ARA-C and polyneuropathy has also been reported with both intravenous [4,5] and intrathecal administration [6]. It can occur after a wide range of doses [7] but is more commonly associated with high doses [8].

Predominantly sensory peripheral neuropathy is seen with low dose ARA-C, whereas sensorimotor neuropathy occurs after high-dose therapy. Symptoms can start from hours to 2–3 weeks after initiation of therapy [6,9-11] and can take several forms. A pure sensory or sensorimotor neuropathy is most common. With higher doses it can also produce both myelopathy and a rapidly progressive ascending motor neuropathy which is typically monophasic causing severe motor weakness, even quadriparesis requiring ventilatory support. [6]. Indeed, high-dose ARA-C has been reported to cause peripheral neuropathies resembling both Guillain-Barré syndrome (as in our patient), brachial plexopathies, and lateral rectus palsies [9-11]. The overall incidence of demyelinating polyneuropathy has been reported to be 1% [6]. The mechanism of the neuropathic effect is unknown. ARA-C incorporates into DNA and inhibits DNA polymerase and, thus, blocks DNA synthesis. In the rapidly progressive form associated with high-dose therapy, autopsy has showed demyelination with intact axons, raising the possibility of a direct neurotoxic effect on Schwann cells. Electrophysiologic studies demonstrate features consistent with both axonal and demyelinating neuropathy [12,13]. This is consistent with nerve biopsy results, which have shown axonal swelling and segmental demyelination [8]. Openshaw reported demyelination identified in luxol-fast blue sections of peripheral nerve with Bielschowsky-stained sections showing intact peripheral nerve axons on autopsy studies [6].

Although central nervous system involvement is the most common neurotoxicity associated with intrathecal methotrexate therapy [9,14,15], transverse myelopathies [16] and motor paraplegias have also been reported (mostly in the pediatric literature) [1,17]. Anderson and colleagues reported on a three-year old girl with ALL who in remission developed lower extremity paraparesis and areflexia 15 days after receiving intrathecal methotrexate, cytarabine, and hydrocortisone. Spinal fluid, imaging, and electrodiagnostic testing was consistent with a polyradiculoneuritis and it was postulated that these findings could represent selective ventral nerve root vulnerability to intrathecal chemotherapy [18]. Rolf and colleagues reported on two pediatric patients with acute lymphoblastic leukemia who developed ascending motoric paraplegia (AMP) following intrathecal chemotherapy. Both patients suffered from progressive weakness of their lower extremities, and it was felt that there was convincing evidence that AMP is caused by spinal cord toxicity of intrathecally applied toxic agents such as cytarabin and/or methotrexate leading to spinal demyelination as demonstrated by elevated myelin basic protein in cerebrospinal fluid [1].

Werner [17] reported the following: Transient or permanent paraplegia after the use of intrathecal (IT) methotrexate (MTX) or cytosine arabinoside (ARA-C) for treatment or prophylaxis of patients with meningeal leukemia is an unusual complication, with an incidence of less than 3% among such patients. Only 15 cases involving IT MTX have been documented and even fewer with IT ARA-C. Three patients were studied who developed permanent or ascending myelopathy from treatment of their leukemia or rhabdomyosarcoma with IT chemotherapy. The patients' ages ranged from 7 to 62 years. Two of the three patients had electromyographic examinations. These revealed a primary motor neuron degeneration or a polyradiculopathy, superimposed on a mild axonal peripheral neuropathy associated with vincristine therapy. This is consistent with other electromyographic studies. Two of the patients showed an elevation of the cerebral spinal fluid (CSF) protein before development of paraplegia; one also showed a rise in myelin basic protein associated with his myelopathy. Neuropathologic findings suggest demyelination as the primary process leading to myelopathy. Increasing evidence has shown that total CSF protein, or more specifically, the myelin basic protein, may be elevated before development of paraplegia. Routine serial testing of the CSF for total protein was suggested as a screening test during therapy.

It should also be mentioned that GBS can be associated with leukemia alone. Vembu and colleagues [19] reported a case of severe GBS in a 32-year old female patient diagnosed with acute lymphoblastic leukemia who was on chemotherapy. The clinical features, nerve conduction and the cerebrospinal fluid studies were consistent with acute GBS and it was felt that the fulminate neuropathy was most likely due to the association between GBS and leukemia rather than chemotherapeutic neurotoxicity as the patient responded to IVIG.

The patient presented developed a third cranial nerve palsy which then progressed to a bilateral facial nerve palsy. At first it was felt that this may be a vasculitis with multifocal involvement. However, given the setting of her motor quadriplegia, respiratory failure along with the vasculitis-negative sural nerve biopsy results it was most likely a manifestation of AIDP or a GBS-like syndrome. Facial paresis, usually bilateral, is found in at least one half of patients [2] and other cranial nerve involvement has also been reported. Given the time course it was felt that the combination of the intravenous ARA-C with the intrathecal methotrexate caused her ascending sensory motor polyradiculoneuropathy with motor quadriplegia, perhaps by selective ventral nerve root vulnerability to intrathecal chemotherapy as has been suggested by Anderson and colleagues [18]. There was no evidence of myelopathy via MR imaging and the electrodiagnostic studies were consistent with diffuse acute axonal loss. The negative cytology results in the CSF with multiple lumbar punctures makes diffuse neoplastic meningitis less likely. Nerve biopsy confirmed predominately axonal loss changes with a secondary drop out of myelinated fibers, and muscle biopsy revealed angulated small fibers consistent with neurogenic atrophy. Unfortunately she did not respond to plasma exchange. It is puzzling why there was never any elevation of the spinal fluid total protein even with two samples taken two weeks apart. There was however on both spinal fluid samples elevated OCB's and MBP as has been previously reported [1,17,20]. Lastly, a direct effect from the AML was felt to be unlikely because the biopsy didn't reveal any leukemic cell infiltration or perilymphocytic infiltrates.

## Conclusion

Although there have been occasional pediatric case reports, this is an interesting case of ascending sensory motor polyradiculoneuropathy with cranial nerve involvement in an adult patient with AML following intravenous ARA-C and intrathecal methotrexate. The polyradiculoneuropathy appears to have been secondary to predominant axonal loss rather than demyelination as reported in most other studies.

## Competing interests

The author declares that he has no competing interests.

## Authors' contributions

RAR wrote the entire manuscript.

## Consent

Written informed consent was obtained from the patients' husband for publication of this case report and accompanying images. A copy of the written consent is available for review by the Editor-in-Chief of this journal.
